# A case of decompression illness not responding to hyperbaric oxygen

**DOI:** 10.1186/s40560-018-0299-3

**Published:** 2018-05-21

**Authors:** Asadullah Naqvi, Derrick Clarence

**Affiliations:** 1 ACCS-ST2 Anaesthetics and Intensive Care Medicine, Walsall Manor Hospital, Walsall, UK; 2 Walsall Manor Hospital, Walsall, UK

**Keywords:** Decompression sickness, Nitrogen narcosis, Diving syndrome, Hyperbaric oxygen, Arterial gas emboli

## Abstract

**Background:**

The case reinforces the importance of stepping back and looking at every possibility along with multiple co-existing pathologies. It takes into account the thought process of multiple systems and a multidisciplinary team approach. Learning points to take are that decompression illness can present atypically, but one must exclude other causes.

**Case presentation:**

We present the case of a 42-year-old male from the West Midlands, UK, who attended the emergency department post-scuba diving with confusion, light-headedness, left arm weakness, and bilateral paraesthesia of the hands. Post-diving, he displayed typical symptoms of decompression illness. He attended the hyperbaric decompression chamber before attending the emergency department but to no resolve. A computed tomography of the head showed no signs of intracranial pathology. He had another session in the hyperbaric oxygen chamber but to no success. Upon admission, his blood showed polycythaemia. His saturation had dropped to 91% on room air, and a computed tomography pulmonary angiogram revealed no obvious cause. A magnetic resonance imaging of his head revealed some deep periventricular ischaemic changes, old and new, however no signs of gas embolism or poor flow. A bubble echo confirmed a patent foramen ovale. A leptospirosis and a vasculitis screen were both negative. Symptoms had slowly improved but he was left with a left arm motor weakness, and the team was left puzzled as to what could have caused his signs and symptoms. Through a diagnosis of exclusion, decompression sickness was the conclusive diagnosis. The patient made a full recovery.

**Conclusions:**

Decompression illness results as a sudden decrease in pressures during underwater ascent; it is caused by nitrogen bubbles forming in tissue. Additionally, a patent foramen ovale allows arterial gas emboli to cause further harm. Type 2 decompression sickness is the more severe form and includes neurological, respiratory, and cardiovascular symptoms.

## Summary

We present the case of a 42-year-old male post-scuba diving who presented to the emergency department with confusion, light-headedness, left arm weakness, and bilateral paraesthesia of the hands. He attended the hyperbaric oxygen chamber but to no resolve. Admission blood showed polycythaemia. His saturations had dropped to 91%. A magnetic resonance imaging revealed some deep periventricular ischaemic changes. A bubble echocardiogram was positive. He was left with a persistent left arm motor weakness. Type 2 decompression sickness (DCS) was diagnosed.

The case reinforces the importance of looking at all differential diagnosis. It takes into account the thought process of a multidisciplinary team approach including intensivists, neurology, and cardiology to name a few. Learning points to take from this case are the differentials, physics of gases, and the mechanism of decompression sickness along with treatment.

## Background

DCS usually responds well to hyperbaric oxygen recompression. It is rarely associated with polycythaemia and desaturation. Serious implications occur when central nervous system symptoms are present and usually indicate a poor prognosis. It requires a multimodal and multidisciplinary approach. We present a case of an experienced 42-year-old male who presented with signs and symptoms of DCS which did not respond well to recompression in the hyperbaric oxygen chamber.

## Case presentation

A 42-year-old male presented to the emergency department (ED) post-scuba diving in a quarry. He has a past medical history of depression, an ex-smoker, drinks 32 units of alcohol per week, in a same-sex marriage, and a body mass index of 39.

He had 2 weeks ago been abroad scuba diving multiple times out at sea at depths of 40 m, after which he flew back to the United Kingdom (UK). A day prior to attending the ED, he had dived three times to depths of 40 m for 30 min each time in a quarry in the UK. Shortly after diving, within 30 min, he experienced symptoms of left arm weakness, confusion, pain in the left ear, and bilateral paraesthesia of the hands. He was taken to a hyperbaric oxygen recompression chamber (HBOC) in Rugby, after which he showed signs of improvement.

He was discharged home by the diving doctor requiring only some assistance with walking. The next morning, he became confused, agitated, feverish, and clammy. He was brought to the ED where his Glasgow Coma Score was 13 and he had a left upper limb weakness. A computed tomography (CT) was conducted of the head. His haemoglobin was 222 g/L, haematocrit was 63%, white cells 28 × 10^9^/L, neutrophils 23 × 10^9^/L, C-reactive protein 81 mg/L, and a mild acute kidney injury. He was haemodynamically stable and was commenced on 1000 ml of 0.9% sodium chloride over 1 h, followed by a maintenance of 3 L over 24 h. After discussion with the diving medicine doctor, he was taken to a HBOC for the second time, but to no avail.

He was admitted and treated for an ischaemic stroke and meningitis with empirical antibiotics. A magnetic resonance scan (MRI) was conducted followed by vasculitis screen. During his stay, his oxygen saturations dropped to 91% on room air and a sinus tachycardia of 120 beats per minute with a blood gas analysis showing a type 1 respiratory failure. A CT-pulmonary angiography was performed, after which his saturations had self-resolved. A bubble echocardiogram was conducted after identifying a heart murmur. Lumbar punctures were later taken as symptoms persisted. Blood cultures were also taken.

On day 2 of admission, his confusion had resolved; he was left with a residual left side arm weakness 4/5 power and dysmetria on the same side. He was advised to never scuba dive again.

## Discussion and conclusion

**Fig. 1:**
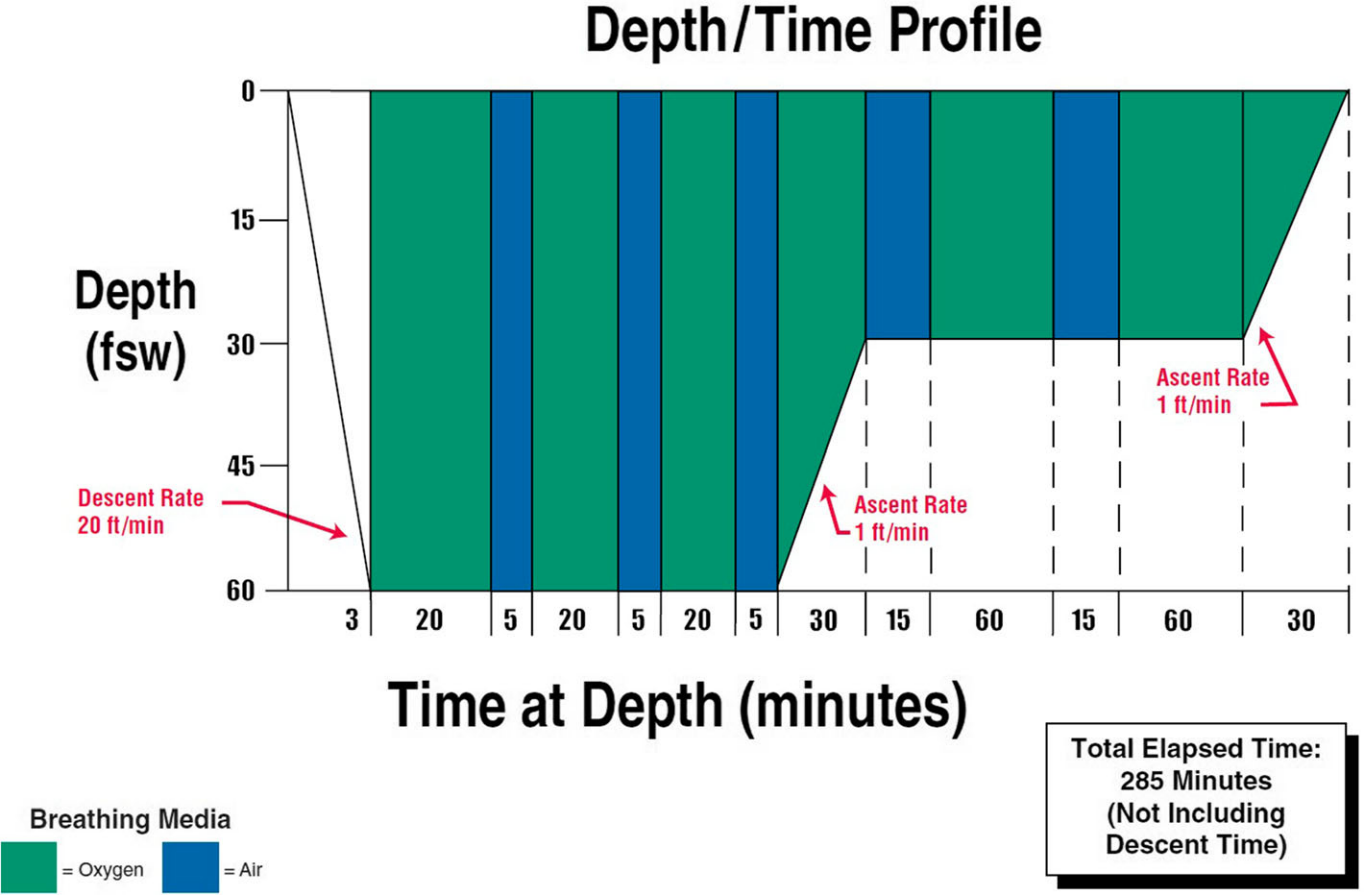
The ‘Royal Navy Treatment Table 62’ for decompression sickness

**Fig. 2:**
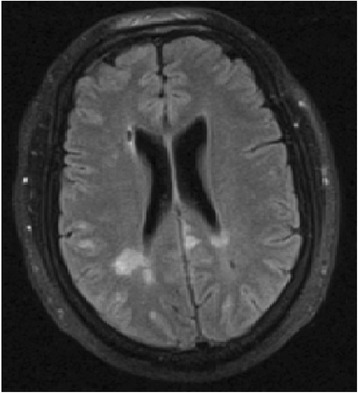
T1-weighted MRI scan showing cortical white matter and subcortical and deep periventricular small ischaemic lesions of old and new age

In this case, it was presumed he had signs and symptoms of decompression illness (DCI) and the patient underwent treatment in the HBOC using the ‘Royal Navy Treatment Table 62’ (Fig. 1) [1] recompression protocol. Due to failure of his response to the HBOC, a stroke was considered to explain his neurological symptoms; however, imaging was negative for an intracranial bleed and he then revisited the HBOC for the same recompression protocol, which had no effect. After reviewing the raised blood inflammatory markers, meningitis was treated for using broad-based antibiotics. In the absence of a positive lumbar puncture for infective meningitis, multiple sclerosis was also tested for which was negative. An MRI of the head revealed cortical white matter and subcortical and deep periventricular small ischaemic lesions of old and new age (Fig. 2). It was suggested by the radiologist that the images are consistent with a vasculitis disease. A vasculitis screen followed and was negative. As the patient then developed sinus tachycardia and with low oxygen saturations, a pulmonary embolism was imaged for by CTPA which was negative. A cardiologist later picked up a murmur on examination, and a bubble echo was conducted which revealed a patent foramen ovale (PFO). This was not a convincing cause for the MRI findings as the location of the lesions were not typical for a gas embolism.

By the advice of a microbiologist, leptospirosis was tested for due to its link with diving in quarries where animal urine may infect humans, this was negative. After 2 days of hospital stay, he had improved and diagnosed with DCS type 2; although left with some residual symptoms, the cause of his recovery was unknown.

DCI results from a change in barometric pressure. As a diver receives oxygen mixed with nitrogen at high pressure during deep dives, nitrogen pressure exceeds that in the blood and dissolves into all body tissues, in particular lipids. At 150 ft, alterations in reasoning, memory, response time, and overconfidence are affected [2]. Nitrogen interferes with the electrical components of the cellular membrane of the nervous system leading to an anaesthetic effect [3].

As a diver begins to ascend, the partial pressure of nitrogen decreases slowly, leaving the body via the lungs. During a quick ascent, the nitrogen changes from liquid to gas state before it leaves the body resulting in bubbles. If a person flies too soon after diving, this additional decrease in cabin pressure which is less than at sea level may further precipitate bubble formation. These bubbles can be harmful depending on their size, quantity, reactions, and locations. Furthermore, the presence of a PFO can allow bubbles to enter the arterial circulation, travelling to organs, resulting in an arterial gas embolism.

Bubbles affect the body in multiple ways, one of which by reduced perfusion that can lead to organ ischaemia in small vessels, as well as causing mechanical compression and stretching of the blood vessels and nerves. Through adhesion-molecule-mediated endothelial activation, bubbles cause platelet and neutrophil activation which further can cause clotting and ischaemia [4]. These cells release micro-particles upon activation and cell apoptosis [4]. Once bubbles form, they create a foreign body interface to which platelets adhere. In severe DCI, significant reduction in platelet count has been identified [5].

In a study, it was found that Von Willerbrand Factor was 2.4 to 11.7 folds higher in patients with decompression sickness [5]. Another study which looked at tropical divers found that a single dive was sufficient to increase haematocrit significantly.

DCI has been divided into two disease types, DCS and AGE. DCS has two types.

Type 1 is a milder form and the most common, which results in mild pains anywhere in the body and often resolve within 10 min, skin manifestation of pruritus and/or cutis marmorata, and most commonly, pains in the joints known as ‘the bends’. Cutis marmorata is hypothesised to be a result of gas bubbles embolising in the brain stem and usually indicates a fine line between types 1 and 2 DCS [6].

Type 2 DCS is a more severe form as it usually includes one or more of pulmonary symptoms, hypovolemic shock due to fluid shift from intra- to extra-vascular space, and nervous system involvement, primarily the spinal cord [7]. Nervous system involvement can include paralysis, paraesthesia, and loss of sphincter control, nausea and vomiting. AGE results from bubbles entering the arterial circulation via a PFO leading to an AGE and organ ischaemia. Patients who have an onset of symptoms within 30 min have vertebral back pain or respond poorly to the HBOC usually have worse outcomes [7].

The management of decompression sickness is with the utilisation of a recompression in a HBOC. The chamber exerts a pressure on the patient imitating commonly a depth of 18 m with 100% oxygen. This allow gases in the body to re-enter soluble form; as the chamber pressure is reduced slowly over several hours, it allows the dissolved gases, in particular nitrogen, to leave the body via the pulmonary circulation and through the lungs. The 100% oxygen is administered with intermittent air to reduce the chances of oxygen toxicity. Most commonly divers respond well to this treatment; in cases where they do not, it is feared symptoms are more severe than perceived. In a study, it was found that up to 6.6% of patients do not respond to the HBOC [8]. Along with the HBOC, blood thinners such as aspirin are given to prevent strokes; however, there are no studies to show its significance [9]. Rehydration is vital as there is usually a degree of dehydration in these patients.

A solution to the nitrogen narcosis and gas bubbles in divers is the use of mixtures where nitrogen is replaced by helium for dives that are deep and prolonged [10]. Helium is less dense than nitrogen; therefore, breathing is less laboured and it leaves the body quicker hence the risk of bubble formation is less, however can still cause narcosis in high concentrations.

In conclusion, acute DCI is a rare presentation and is a clinical diagnosis that requires a fair amount of clinical suspicion. Due to its rarity, clinicians may have difficulty in management of this condition and its differentials, therefore making it an important discussion amongst the field of intensivists. Because symptoms can vary dramatically, other possible causes must be investigated. In most cases, diagnosis is made when recovery is seen through HBOC. No specific tests exist for DCI, but a bubble echo can assist with understanding the disease type.

## Abbreviations

CT: Computed tomography
DCI: Decompression illness
DCS: Decompression sickness
ED: Emergency department
HBOC: Hyperbaric oxygen chamber
MRI: Magnetic resonance imaging
PFO: Patent foramen ovale
UK: United Kingdom
